# Exosome-Derived microRNA: Efficacy in Cancer

**DOI:** 10.7759/cureus.17441

**Published:** 2021-08-25

**Authors:** Jaskamal Padda, Khizer Khalid, Anwar Khedr, Vinay Patel, Ola A Al-Ewaidat, Fahriba Tasnim, Sandeep Padda, Ayden Charlene Cooper, Gutteridge Jean-Charles

**Affiliations:** 1 Internal Medicine, JC Medical Center, Orlando, USA; 2 Internal Medicine, Advent Health & Orlando Health Hospital, Orlando, USA

**Keywords:** microrna, cancer, tumor suppressor, oncogene, cancer screening

## Abstract

Exosome-derived microRNA (miRNA) has been the focus of attention in recent years. Mainly, their role in the pathogenesis of different types of cancer has been extensively studied. The different types of exosomal miRNAs (exomiRs) act as either oncogenes or oncosupressors. They have potential prognostic and diagnostic efficacy in different types of cancer due to their high stability and easy detection in bodily fluids. This is especially true in lung cancer, colorectal cancer, ovarian cancer, and breast cancer. However, their efficacy as potential therapies has not been widely investigated. This review will discuss the structure and functions of exosomes and miRNA, as well as the role of exomiRs in the pathogenesis of different types of cancer through boosting growth, promoting progression, chemotherapy resistance, angiogenesis, metastasis, and immune system evasion. We will also discuss the application of exomiRs in diagnosing different types of cancer and their role in prognosis. Furthermore, we shed light on the challenges of developing therapeutic agents using miRNAs and how the carriage of therapeutic miRNA by exosomes can help solve these challenges. Finally, we examine recent studies exploring the potential of exomiRs in treating cancers such as neuroblastoma, glioblastoma, and melanoma.

## Introduction and background

In 2015, cancer was the second leading cause of mortality in the United States, with about 13% of deaths resulting from cancer worldwide. The mortality rate of certain cancers has dropped recently, probably due to the advancement in screening methods and treatment measures. However, cancer incidence has remained the same [1], which has led to increasing efforts in trying to find more feasible and effective screening biomarkers and novel therapeutics for cancer. Exosomal microRNAs (exomiRs) have been extensively studied in this regard [2].

Exosomes are small extracellular vesicles that are secreted by all cell types in the human body. They act as an intercellular communication method. They contain DNA, RNA, proteins, and lipids. Cancer cells tend to secrete increased amounts of exosomes than normal cells, which allows them to be considered as candidates for future cancer screening biomarkers [3]. MicroRNAs (miRNAs) are small, noncoding RNA molecules that act as significant regulators of gene silencing. They represent less than 5% of the human genome and regulate about one-third of human protein-coding genes. Dysregulation of miRNAs has been detected in cancer cells. They act as tumor suppressors and oncogenes, for which they have been studied as promising cancer diagnostics and therapeutics [4,5]. In this article, we will discuss exosomes and miRNAs in detail, the role of exomiRs in cancer pathogenesis, and their application as potential diagnostic and prognostic cancer biomarkers. In addition, we will review their efficacy as potential cancer therapeutics and the reported challenges.

## Review

What are exosomes?

Exosomes are 30-200 nm small, single-membrane organelles that have the same topology as the cell. Exosomes are filled with lipids, proteins, nucleic acid, and glycoconjugates [6]. They are extracellular vesicles that play a significant role in intercellular communication. Cancer exosomes carry malignant information which can be used to reprogram recipient cells [7]. They work to promote a favorable microenvironment that supports tumor growth and prevents apoptosis. Cancer exosomes have the ability to form new vessels which provide nutrients, oxygen, and waste removal, as well as contribute to the metabolic reprogramming of cancer cells for their sustained proliferation [7]. They are isolated from bodily fluids and blood [8]. Exosomes are created by budding at the endosome and plasma membranes. They contain a wide variety of protein complexes and show high molecular heterogeneity. Exosomes help remove unnecessary cellular proteins from the cell and act as intracellular messengers because they fuse easily with membranes of neighboring cells [9]. The functions of these exosomes are diverse, including signal molecule transfer from cell to cell and extracellular matrix remodeling. Immunity, cancer, tissue homeostasis, neurodegenerative disease, and human development are aspects of human health that exosomes impact [6]. Due to these properties, exosomes are being developed as therapeutic agents in multiple diseases, including cancer treatment by modulation of the immune response, metastasis, and tumor microenvironment reprogramming [6-8].

What are microRNAs?

Definition and Function

miRNAs are short sequences (21-25 nucleotides) of single-stranded RNA molecules. Although they are noncoding RNA molecules, they play a vital role in regulating gene expression post-transcriptionally through different methods [5]. Mainly, they bind to the 3' untranslated region of the target messenger RNA (mRNA) to induce its degradation and inhibit translation. However, they also bind to the 5' untranslated region of the mRNAs, gene promoters, and coding sequences. This reveals their additional role in translation activation and transcription regulation under certain conditions [10]. The role of miRNAs in regulating gene expression has made them a pivotal regulator of different biological events in the human body, such as cell proliferation, differentiation, and apoptosis. In addition, they regulate the homeostasis of iron, glucose, and cholesterol along with immune system regulation [11].

Biogenesis and Isolation

RNA polymerase II transcribes miRNA genes in the nucleus, producing a single-stranded RNA molecule called primary miRNA. Primary miRNA forms a hairpin loop structure to stimulate a nuclear ribonuclease complex called Drosha/DiGeorge syndrome critical region 8. This complex cleaves primary miRNA and produces a precursor miRNA that is transferred to the cytoplasm by the nuclear transporter factor Exportin 5/RanGTP [12]. In the cytoplasm, precursor miRNA undergoes further cleavage by ribonuclease III endonuclease Dicer and produces mature miRNA which binds to the Argonaute protein-producing, RNA-induced silencing complex. The RNA-induced silencing complex guides the miRNA regulatory function by binding it with the target mRNA molecules through direct base pairing [13]. The degree of this base pairing determines the way of gene silencing either through mRNA degradation or translation inhibition for an extensive and limited degree of base pairing [5]. miRNAs are secreted selectively in the extracellular fluid. Extracellular miRNAs (ECmiRNAs) have been detected as stable molecules in the blood, different biological fluids, and cell cultures. Their extracellular space stability is attributed to their packaging proteins and membranous particles, which protect them from ribonuclease activity. This makes these molecules promising biomarkers for different diseases. However, the main challenge in the isolation of ECmiRNAs is their short sequence and low amount in the extracellular fluid. In addition, there are different variables that can affect the efficiency of the isolation of ECmiRNAs. These include the sample collection method (whole blood versus plasma-based), extraction method (phenol/chloroform-based extraction versus column-based extraction method), and quantification method (quantitative reverse transcription-polymerase chain reaction [RT-qPCR] versus microarrays) [14]. Therefore, a standardized protocol is needed to optimize the isolation of miRNAs.

Role of exosomal microRNA in cancer pathogenesis

**Figure 1:**
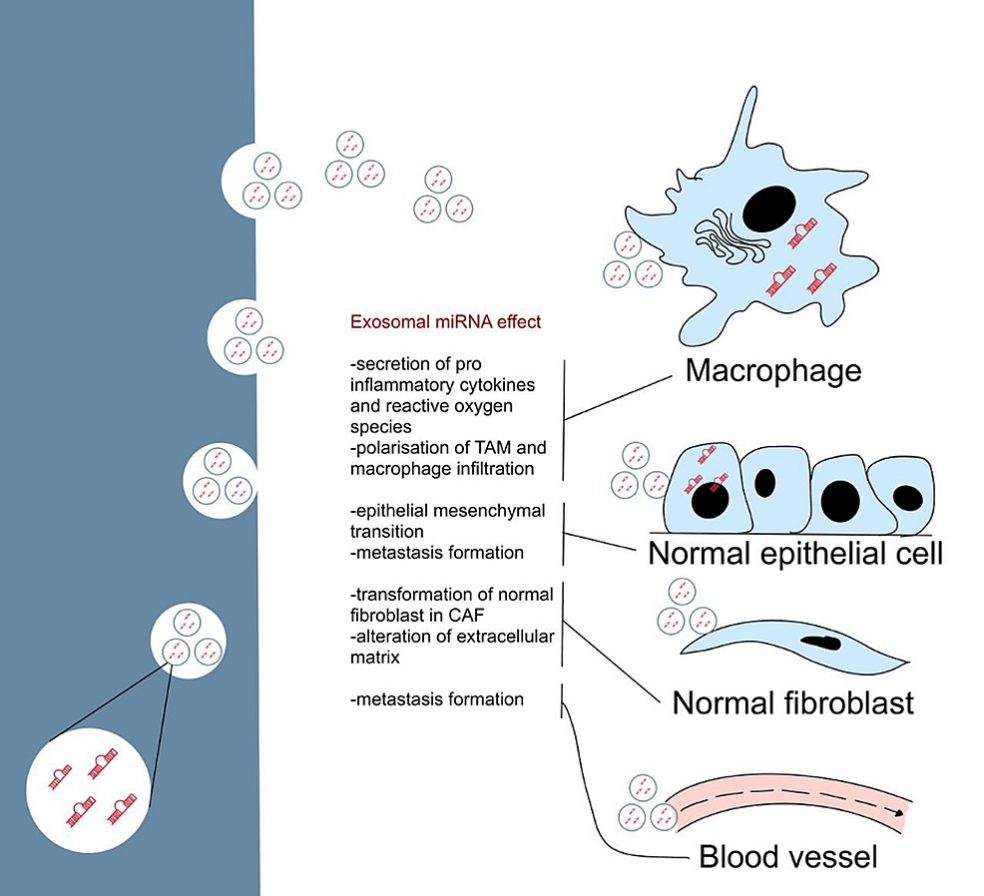
Pathogenic mechanism of exosomal miRNAs. miRNA: microRNA; TAM: tumor-associated macrophage; CAF: cancer-associated fibroblasts Copyright/License: Licensee MDPI, Basel, Switzerland. This figure is from an open-access article distributed under the terms and conditions of the Creative Commons Attribution (CC BY) license (http://creativecommons.org/licenses/by/4.0/). Adaptations were made to the original figure. Ingenito F, Roscigno G, Affinito A, Nuzzo S, Scognamiglio I, Quintavalle C, Condorelli G: The role of exo-miRNAs in cancer: a focus on therapeutic and diagnostic applications. Int J Mol Sci. 2019, 20:4687 [3].

Exosomes are involved in transferring miRNA from cell to cell like a messenger. This communication helps in the reprogramming of target cells in terms of invasion, gene expression, tumor growth, angiogenesis, and immune function. Figure 1 shows the pathogenic mechanism of exomiRs [3]. In a study, exomiR has been shown to support tumor growth and inhibition of apoptosis via loss of phosphatase and tensin homolog [15]. miR-660-5p has been shown to promote tumor proliferation and survival by targeting Krueppel-like factor 9 in nonsmall cell lung cancer (NSCLC) [16]. One exomiR can impact different cancer aspects at the same time. In breast cancer (BC), miR-155 has been shown to induce drug resistance to paclitaxel and doxorubicin and as an oncogenic signal [17,18].

Angiogenesis has been shown to be affected by exomiRs released from cancer cells. Chronic lymphocytic leukemia has been thought to release miR-21, miR-155, miR-148a, miR-let-7g, and miR-146a, which are taken up by endothelial and mesenchymal cells [19]. A lung cancer study has shown the transfer of miR-21 via exosomes, which is taken up by endothelial cells [20]. Distant organ invasion and extravasation, intravasation, and migration in the vascular system involve complex processes. ExomiRs released from cancer cells can enhance tumor cell migration and manipulate the local stoma of tissue to facilitate metastasis. The Mir-200 family of miRNA, which are considered to be tumor suppressor genes, can be transferred from metastatic cells to nonmetastatic cells. This promotes metastatic capabilities in weak metastatic cells giving them the ability to colonize distant tissues [21]. Some of these oncogenic and tumor-suppressor miRNA and their effect on various cancers have been shown in the tables below. Table 1 demonstrates the effects of oncogenic miRNA in tumor progression, while Table 2 shows the tumor-suppressor miRNA effects of tumor progression [3].

**Table 1:** Oncogenic miRNA. ↑: increase; ↓: decrease: miR: microRNA, EMT: epithelial-mesenchymal transition Copyright/License: Licensee MDPI, Basel, Switzerland. This figure is from an open-access article distributed under the terms and conditions of the Creative Commons Attribution (CC BY) license (http://creativecommons.org/licenses/by/4.0/). Adaptations were made to the original table. Ingenito F, Roscigno G, Affinito A, Nuzzo S, Scognamiglio I, Quintavalle C, Condorelli G: The role of exo-miRNAs in cancer: a focus on therapeutic and diagnostic applications. Int J Mol Sci. 2019, 20:4687 [3].

Exosomal miRNA	Effect	Tumor
miR-10b	Invasion ↑	Breast cancer
miR-32-5p	Drug resistance ↑	Hepatocellular carcinoma
miR-223	Invasion ↑	Pancreatic cancer
miR-9	Angiogenesis ↑	Glioma
miR-99a-5p, miR-125b-5p	Drug resistance ↑	Large B-cell lymphoma
miR-21, miR-378e, miR-143	EMT and stemness ↑	Breast cancer
miR-93-5p	Growth ↑	Esophageal cancer

**Table 2:** Tumor-suppressor miRNA. ↑: increase; ↓: decrease; miR: microRNA Copyright/License: Licensee MDPI, Basel, Switzerland. This figure is from an open-access article distributed under the terms and conditions of the Creative Commons Attribution (CC BY) license (http://creativecommons.org/licenses/by/4.0/). Adaptations were made to the original table. Ingenito F, Roscigno G, Affinito A, Nuzzo S, Scognamiglio I, Quintavalle C, Condorelli G: The role of exo-miRNAs in cancer: a focus on therapeutic and diagnostic applications. Int J Mol Sci. 2019, 20:4687 [3].

Exosomal miRNA	Effect	Tumor
miR-192	Metastasis ↓	Lung adenocarcinoma
miR-9	Angiogenesis ↓	Nasopharyngeal carcinoma
miR-451a	Apoptosis ↑, angiogenesis ↓	Hepatocellular carcinoma
miR-8073	Growth ↓	Colorectal cancer
miR-16-5p	Growth ↓, migration ↓, invasion ↓	Mesothelioma
miR-100	Angiogenesis ↓	Breast cancer
miR-199a	Growth ↓, migration ↓, invasion ↓	Glioma

Potential applications of exosomal miRNA as diagnostic and prognostic markers in cancers and their efficacy

Lung Cancer

Lung cancer is the most common cause of cancer-related death in both men and women worldwide [22]. Unfortunately, due to the asymptomatic nature of its early stages, it is often diagnosed at a late stage and results in poor treatment and survival outcomes. Therefore, it would be of immense benefit if lung cancer can be diagnosed early using noninvasive molecular markers [23].

Among studies that aimed to identify circulating exomiRs, a study by Rabinowits et al. [24] was significant as the authors documented notable upregulation of eight different miRNAs in the exosomes of NSCLC patients as opposed to healthy individuals. Later, Cazzoli et al. [25] could differentiate lung adenocarcinoma from healthy controls and patients with granulomas using specific panels of four exomiRs (miR-378a, miR-379, miR-139-5p, and miR-200b-5p) and six exomiRs (miR-151a-5p, miR-30a-3p, miR-200b-5p, miR-629, miR-100, and miR-154-3p), respectively. Additionally, Jin et al. [26] identified upregulations of exomiRs that were specific to squamous cell carcinoma patients. Several other studies have also demonstrated upregulation of exomiRs in patients with lung cancer [27-35]. Roman-Canal et al. [36] identified an increased level of exomiRs in pleural and lavage fluid in patients with lung adenocarcinoma.

Moreover, the study by Boeri et al. [37] was able to define signatures of circulating miRNAs with predictive potential in lung cancer growth. They distinguished 15- and 9-miRNA signatures in pre-disease plasma samples, which had the potential to predict high risk for lung cancer development and to distinguish aggressive and early metastatic tumors, respectively. Hence, exomiRs can be used not only for diagnostic purposes but also in population screening trials.

When it comes to prognosis, several studies have demonstrated that higher levels of exomiRs correspond with poor survival [38-44]. Even following curative surgery and adjuvant chemotherapy (for stages II/III in NSCLC), higher levels of miR-451a were identified as an independent predictor of disease recurrence and poor survival [45]. Moreover, increased levels of exomiR-96 have been linked with advanced tumor stage and grade as well as lymph node metastasis in lung cancer patients, while it was also discovered that disease progression was regulated by exomiR-96 through targeting the tumor-suppressor LIM-domain-only protein 7 [46].

Colorectal Carcinoma

The main tests for screening and diagnosing colorectal carcinoma (CRC) are colonoscopy, flexible sigmoidoscopy, and fecal-based tests. Unfortunately, it is hard to use these tools for general population screening as they are either invasive and costly or have poor sensitivity and specificity [47,48]. Hence, a noninvasive molecular marker such as exosome-derived circulating miRNAs for early diagnosis and prognosis has become a promising area of research [49,50]. Ogata-Katawa et al. [51] were the first to report seven subtypes of miRNAs to be upregulated in CRC patients as opposed to healthy individuals using RT-qPCR validation and microarray profiling. Tsukamoto et al. [52] also supported elevated exomiR-21 level in CRC as they reported a similar finding in their study. In addition, several other studies have also reported upregulation of exomiRs in CRC [53-55].

In addition to diagnosis, exomiRs have also proven to be a potential prognostic biomarker of CRC. For example, Takano et al. [56] reported that an increased level of exosomal miR-203 was seen to be linked to a higher risk for liver metastasis and poor survival outcome. Furthermore, elevated exomiR-19a [57] and miR-6803-5p [58] levels were linked to advanced Tumor-Node-Metastasis staging, lymph node and liver metastasis, and poor survival. Several other studies demonstrated that some other exomiRs had the potential to indicate worse survival expectancy [54,59].

Breast Cancer

Exosome-derived miRNAs have emerged as a significant biomarker in BC diagnosis and clinical management [60,61]. Elevated levels of miR-1246 and miR-21 plasma exosomes have been demonstrated to differentiate BC patients from healthy controls [62,63]. Furthermore, upregulation of exosomal miR-223-3p, miR-16, miR-27a/b, miR-152, miR-199a-3p, miR-340, miR-376a, miR-410, and miR-598 along with downregulation of miR-30c and miR-150 can be suggestive of the patient having breast tumors compared to healthy women [64-66]. Interestingly, elevated serum exomiR-21 levels can differentiate metastatic from nonmetastatic breast tumors [67]. Moreover, serum exomiR-373 has been linked to aggressive human epidermal growth factor receptor 2+ and triple-negative BC [68], whereas miR-16 levels have been linked to estrogen receptor+/progesterone receptor+ BC [65].

Ovarian Cancer

Circulating miRNAs have also proven to have diagnostic potential and can be used as a supportive tool in diagnosing ovarian cancer (OC) [69,70]. Taylor and Gercel-Taylor [71] were the first to report the diagnostic value of exomiRs in OC and identified upregulation of eight specific miRNAs in the serum exosomes of OC patients compared to healthy women. The concentration of miR-200a/b/c was found to be the efficient one that could distinguish ovarian tumors from benign ovarian lesions. In addition, exomiRs have prognostic potential as increased levels of miR-373 and miR-200b/c have been appreciably linked to lymph node metastasis, advanced International Federation of Gynecology and Obstetrics stages, higher cancer antigen 125 levels, and poor survival [72].

Other Cancers

ExomiRs have also been proven to have diagnostic potential in other cancers. It has been demonstrated that exosomal miR-10b-5p, miR-195-5p, miR-20a-3p, and miR-296-5p levels are significantly upregulated in gastric cancer patients compared to healthy individuals and can have clinical value in the diagnosis of gastric cancer [73]. In the diagnosis of hepatocellular carcinoma, the increased exosomal level of miR-21 has shown great diagnostic and prognostic significance as it can distinguish cancer patients from chronic hepatitis B patients and healthy controls. It has also been linked to advanced tumor stages [74]. Moreover, plasma exosomal miR-21 [75], miR-34a, miR-34b, and miR-34c levels have been verified to be a diagnostic as well as a prognostic tool in hepatoblastoma [76]. In patients with pancreatic ductal adenocarcinoma, the high levels of miR-10b, miR-21, miR-30c, and miR-181a and decreased let-7a levels have been proven to differentiate these patients from healthy individuals [77]. Tanaka et al. [78] have identified significant elevation of exomiR-21 in patients with esophageal squamous cell carcinoma compared to healthy individuals, and it has also been found to correlate with advanced tumor classification and metastasis. In hematologic malignancies, exomiRs have shown great potential. Hornick et al. [79] have demonstrated an increased level of miR-150, miR-1246, and miR-155 in acute myeloid leukemia patients compared to healthy individuals. Concerning melanoma, lower serum exomiR-125b levels were found to be correlated with advanced disease stage compared to healthy individuals [80]. In clear cell renal cell carcinoma, elevated serum exosomal miR-210 and miR-1233 levels were found to have diagnostic value [81], whereas raised levels of plasma exosomal miR-26a-1-3p, let-7i, and miR-615-3p correlated with adverse outcomes in these patients [82]. Regarding prostate cancer, Mitchell et al. [83] found significant upregulation of miR-141 in patients with prostate cancer, proving it to be a potential diagnostic tool. Li et al. [84] have also verified the capacity of increased exomiR-141 levels to distinguish metastatic from localized prostate cancer.

Challenges to utilize microRNAs as therapeutic agents and how exosomes can overcome these challenges

The fundamental role of miRNAs in regulating gene expression and their involvement in different physiological and pathological events in the human body, particularly tumorigenesis, make them a target for different disease therapeutics [3]. Despite the stability of miRNAs in extracellular fluids, its integration as an effective therapeutic agent has been challenging [85]. The major challenge in developing a novel miRNA treatment is their delivery methods and restricted penetration to the target site. To overcome this challenge, multiple molecular transporters have been created and tested to deliver miRNAs effectively. These include liposomes, exosomes, viral vectors, polymers, and conjugation with sugars, lipids, and proteins [86]. However, regardless of the delivery methods used to transfer miRNAs, they tend to have intracellular interactions with acidic endosomal contents, resulting in their degradation by nucleases and further complicating the situation. There have been different protocols to escape endosomes by using pH-sensitive lipoplexes and polyplexes as well as photosensitive molecules [86,87].

In addition, the free naked ECmiRNAs are unstable and susceptible to rapid RNAase degradation. Moreover, they have a short half-life in the blood due to their rapid renal clearance. This can be managed by chemical modification which can further increase the affinity and potency of miRNAs [88]. Because a single miRNA can affect multiple mRNAs, there is a risk of unwanted side effects based on their interactions with nontargeted genes. The simultaneous use of different miRNAs that regulate the same gene has been considered as a way to overcome this obstacle [89]. Another issue is the probable immune system reaction against miRNAs and their delivery molecules. This can be treated by chemical modification and using highly specific structures that bind to the target tissues allowing lower dosage use which further decreases any unwanted side effects [86].

Exosome usage as a carrier of miRNAs has been found to be more effective than other carrier methods. This is because they are naturally occurring molecules allowing the escape of unwanted immune reactions, they can cross the blood-brain barrier, and they can escape the endosomes. Furthermore, they bind specifically to tumor cells which limits any systemic toxicity. However, multiple challenges have been addressed with the use of exosomes as a carrier of miRNA, including the absence of an effective extraction and purification method, their rapid plasma clearance, their low drug loading, and the fact that they may show different properties regardless of their cell of origin [86,90]. Thus, further efforts are needed to study exosomes and their implication in different therapeutics to find ways to overcome these obstacles [90].

Efficacy and prospects of exosome-derived microRNAs’ applications in cancer treatment

Several studies have proved exomiRs to be a potential therapeutic strategy for human cancer as they have significant roles in many cellular processes as well as strong stability, secretion into all biological fluids, and tissue-specific expression [91-94]. Exosomes can inhibit the expression of onco-miRNAs as they can deliver antagonist tumor-suppressive miRNAs, and thus can be used in cancer therapy. Additionally, exosomes can be removed from the circulatory system, or the fusion/uptake of exosomes by target cells can be prevented as a therapeutic strategy to inhibit tumorigenesis. Exosomes can be separated from a patient’s circulatory system, and after modification, they can be relocated to the same patient for cancer therapy [95-97]. Moreover, the greater half-life than liposomes enables exosomes to bind to recipient cell receptors specifically and, subsequently, creates the possibility to generate exosomes that can specifically target a cell type [94].

Recent studies have revealed that tumor-suppressor, miRNA-loaded exosomes can inhibit tumor angiogenesis if used against proangiogenic mRNAs. Exosomes can also be used in genetic therapy by delivering therapeutic genetic agents to target cells in specific disorders [98,99]. Some exomiRs can also be deemed potential candidates to inhibit tumor growth by specific gene knockdown as exosomes can be used as nanovectors, which can deliver targeted anticancer drugs with low immunogenicity and toxicity compared to other drug-delivery systems [100,101]. Schmittgen et al. [102] revealed that miR-186 could be delivered and that its level can be restored using natural killer cell-derived exosomes or nanoparticles. Thus, natural killer-mediated cytotoxicity can be restored, and tumor size can be reduced in neuroblastoma. Moreover, due to the vital role of exosomes in cell-to-cell communication, new experiments aim to inhibit exosome release or uptake. Ras-related protein Rab-27A depletion precedes a decrease in miR-494 abundance and consequently causes decreased tumor growth and metastasis in melanoma [103]. It has also been proven that exosomes can be used to deliver antisense oligonucleotides to impede cell proliferation in leukemia and BC [104]. Another study supported exomiR as a promising therapy in lung cancer as it demonstrated that exosome-derived miR-302b could significantly suppress lung cancer cell proliferation and migration via the transforming growth factor-beta receptor II/extracellular-signal-regulated kinase pathway [105]. Another study revealed that exosomes could be used to effectively deliver antitumor miRNA to cancer tissues in vivo [106]. Moreover, intratumoral injection of exosomes obtained from miR-146-expressing marrow stromal cells substantially decreased glioma xenograft growth in a rat model of a primary brain tumor [107].

Further, these exosomes have caused diminished expression of a multidrug transporter that boosts resistance to glioblastoma multiforme by inhibiting miR-9 and subsequently sensitizing them to temozolomide. Thus, exomiRs have shown significant promise to be used in cancer therapy [108]. Although many clinical trials are ongoing regarding the use of exosome-based cancer therapy, to implement the use of exosomes clinically as cancer therapy, further research and validation are needed.

## Conclusions

Exosome-derived miRNA has been shown to have a complex role in tumor progression. Exosomes are involved in transferring miRNA from cell to cell like a messenger. This communication helps in the reprogramming of target cells in terms of invasion, gene expression and tumor growth, angiogenesis, and immune function. ExomiR has also been found to have diagnostic and prognostic value in various cancers including lung cancer, CRC, OC, and BC. The fundamental role of miRNAs in regulating gene expression and their involvement in different physiological and pathological events in the human body, particularly tumorigenesis, make them a target for different disease therapeutics. Several studies have proven exomiRs to be a potential therapeutic strategy for human cancer as they have significant roles in many cellular processes. Exosomes can also be used in genetic therapy by delivering therapeutic genetic agents to target cells in specific disorders. Free miRNA instability, unwanted side effects due to effect on multiple mRNAs, and immune sensitivity have been a few challenges that have kept miRNA from becoming a mainstay choice in cancer therapy. Although many studies have shown the significant role of exomiR in cancer pathogenesis and treatment target, more research is needed to obtain more validating data on the utility and effectiveness of miRNA as a potential definitive target for cancer chemotherapy in the future.
